# Translating desktop success to the web in the cytoscape project

**DOI:** 10.3389/fbinf.2023.1125949

**Published:** 2023-03-23

**Authors:** Dexter Pratt, Rudolf T. Pillich, John H. Morris

**Affiliations:** ^1^ Division of Genetics, Department of Medicine, University of California San Diego, La Jolla, CA, United States; ^2^ Resource on Biocomputing, Visualization, and Informatics, University of California San Francisco, San Francisco, CA, United States

**Keywords:** cytoscape, network biology, data visualisation, systems biology, bioinformatics, software development

## Abstract

Cytoscape is an open-source bioinformatics environment for the analysis, integration, visualization, and query of biological networks. In this perspective piece, we describe our project to bring the Cytoscape desktop application to the web while explaining our strategy in ways relevant to others in the bioinformatics community. We examine opportunities and challenges in developing bioinformatics software that spans both the desktop and web, and we describe our ongoing efforts to build a Cytoscape web application, highlighting the principles that guide our development.

## Introduction

Cytoscape (Shannon et al., 2003; Smoot et al., 2011) is an open-source bioinformatics environment for the analysis, integration, visualization, and query of biological networks. Since its inception in 2001, the Cytoscape desktop application has served as a standard tool in academia and industry, and as such, it is downloaded about 300,000 times each year.

In recent years, the web has become a popular alternative to access applications and services as compared to local systems. By accessing applications on the web, users are free from the constraints imposed by their hardware and operating systems and from the inconvenience of carrying their work material from one computer to another. Today, the increasing power and sophistication of web technologies have provided us, and many other bioinformatics software developers, opportunities to extend the impact of our tools through an integrated web-based ecosystem. But the web also challenges tool developers to find ways to best harness the distinct capabilities of desktop and web environments. The transition of the Cytoscape desktop application to a web-based application presents a unique set of challenges, as the functionality of Cytoscape is fundamentally different from existing web-based network analysis tools.

Here, we use Cytoscape as a case study to present six principles of our development approach to transition a mature bioinformatics software system to one that spans both the desktop and the web.

## Discussion

In keeping with our long-standing commitment to our users and community-based software development, we discuss the principles that guide our process, design choices, and the decision to build the new “Cytoscape Home” software publication portal as part of the project.

### Identify what makes the application successful

The keys to Cytoscape’s success are that it provides a general-purpose workbench for network analysis and visualization and is extensible by community-developed apps. It operates on a wide variety of networks without commitment to any particular model of biology. Cytoscape is data-centric, with general functionality for network import and export and integrating networks with-omics and other large datasets. It provides high-performance rendering of large networks, rule-based, data-driven visual styles, network layout *via* a large library of layout algorithms, and network filtering and query. Users can perform a wide range of analyses thanks to the rich ecosystems of apps developed by the community and readily accessible *via* the AppStore (Lotia et al., 2013), containing over 300 apps and clocking around 2000 daily downloads. Workflows can be automated *via* the CyREST (Ono et al., 2015) interface that enables the Cytoscape desktop to be used directly by programs and web applications.

When transitioned to the web, Cytoscape functionality will fill a different role than existing resources, such as databases of pathways, pathway editors, or web applications focused on specific analyses.

Many important biological network resources on the web, such as STRING (Szklarczyk et al., 2016), GeneMania (Franz et al., 2018), IntAct (Del Toro et al., 2021), Reactome (Jassal et al., 2019), and WikiPathways (Martens et al., 2021), provide comprehensive views of the protein-protein interactions, molecular interactions, and biological pathways related to sets of genes/proteins. In contrast, Cytoscape is a general-purpose workbench, providing a much broader range of functionality. For example, it provides a general facility for mapping arbitrary data to network nodes and edges, while WikiPathways and STRING focus on integrating a specific kind of data, such as expression fold changes.

Cytoscape shares some functionality with specialized pathway editing tools such as PathwayMapper (Bahceci et al., 2017) and the most recent Newt Editor (Balci et al., 2021). For example, Newt provides drawing, styling, layout, and diagramming capabilities to create small biological pathway models in the SBGN language and to map data to those pathways. It is a focused application committed to a specific representation of biology, in contrast to Cytoscape’s broader mission.

### Determine the most important uses

First and foremost, a software application must continue to excel at its common uses as it expands onto the web and cloud. In the Cytoscape project, a common mode of use is to interpret ‘omics data *via* integration with molecular interaction networks, followed by analysis and network visualization. In these workflows, users create networks from interaction data, annotate the nodes with experimental data such as differential expression fold changes, apply a layout highlighting node relationships and network structure, and present the data using rule-based visual styles.

The essential analyses, such as network clustering and functional enrichment, are evident in the most frequently downloaded Cytoscape Apps (Maere et al., 2005; Bindea et al., 2009; Montojo et al., 2010; Bindea et al., 2013; Wang et al., 2015; Doncheva et al., 2019).

When looking at publications in the Cytoscape Tumblr Archive between January and November 2022 (https://cytoscape-publications.tumblr.com/), we see that 42% of the papers cited Cytoscape because they made use of apps capable of performing specific analyses such as clustering, enrichment, or topological analysis of hub nodes and fragile motifs using the cytoHubba (Chin et al., 2014) app, that just by itself scored a total of 698 citations in 2022 according to PubMed.

### Plan the transition

It must be easy for current users to adopt the web-based software. The Cytoscape desktop interface design has been honed by 20 years of success, and we are therefore adopting a familiar look and feel on the web, immediately recognizable to users. These measures help current users succeed in using web-based Cytoscape functionality without reading documentation. For example, the rule-based visual style editor, or Vizmapper, has a sophisticated interface that will be conserved between the desktop application and the new web version. Another pillar for any workflows in the desktop application is the “session” feature, which enables users to organize their networks for a given project, storing them as a snapshot of the Cytoscape environment. In Cytoscape web, we will introduce a comparable “workspace” feature with a similar user experience to organize a project’s networks in the cloud.

Nonetheless, many users will continue to use the Cytoscape desktop because of its unique features, apps, and high-performance visualization of very large-scale networks. We have therefore continued to support the Cytoscape desktop even as development shifts to web-ready components.

### Design for extensibility and third-party development

In the Cytoscape ecosystem, “app” development is taking many forms, expanding the opportunities for community development. To achieve the extensibility needed to support third-party developers, Cytoscape Web will be a lean substrate on which others can build. It will connect to the world *via* straightforward programming interfaces and data standards, such as our CX (Cytoscape eXchange) network format. Reusable, CX-based components will enable it to receive networks from other web applications or send networks to them. In the longer term, developers will be able to write code modules comparable to the Cytoscape desktop apps: tightly integrated into the web user interface and installed by users from a trusted repository.

Already, web services are a cornerstone of our modular Cytoscape design strategy. For example, network clustering algorithms can run on remote servers that receive networks and return cluster annotations. By publishing the communication protocols, we enable anyone to build compatible services and add new algorithms. Because services run separately from each other, they can be implemented in any language, run on any platform, and evolve independently from the Cytoscape interface. Services are also reusable resources, callable by web apps, scripts, notebooks, or the Cytoscape desktop application.

### Build the core

**FIGURE 1:**
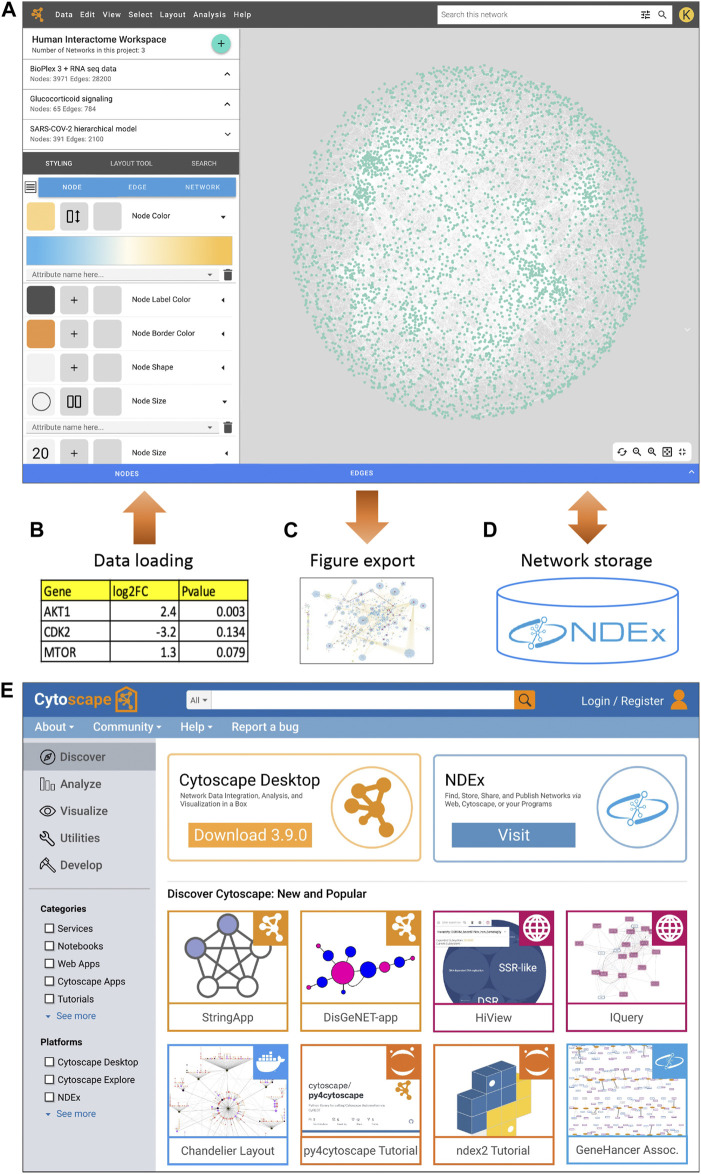
**(A)** A Cytoscape web application concept design, showing the visual style editor and other interface components. **(B)** Loading tabular data to create or annotate networks. **(C)** Exporting a network as a production-quality figure. **(D)** Storing networks in NDEx. **(E)** A Cytoscape Home portal concept design.

In a project such as this, it is important to target an early, robust release of web functionality, deferring the design and migration of advanced features pending an analysis of user feedback and adoption. The initial Cytoscape web system will focus on tools for essential operations such as the style editor to create rules linking visual attributes to data values (Figure 1A), an interface to import data tables to create or annotate networks (Figure 1B), automatic layout tools to set the positions of nodes, and the ability to export the visualized network as a publication-quality figure (Figure 1C). Our existing network data commons on the web, NDEx, the Network Data Exchange (Pratt et al., 2015; Pratt et al., 2017; Pillich et al., 2021), will enable users to store, share, and manage their networks (Figure 1D). Other essential capabilities of the web application will include clustering, functional enrichment analysis, and identification of active subnetworks.

### Catalyze the community

To continue our long-standing community-building strategy on the web, we are expanding the Cytoscape AppStore to become “Cytoscape Home” (Figure 1E). In this portal, developers can register a broad range of web resources along with desktop apps. At Cytoscape Home, users will discover web applications, services, modular software components, and data resources compatible with Cytoscape Web.

## Conclusion

We are expanding on the success of the Cytoscape desktop application by keeping a tight focus on the essential value of Cytoscape to its users, designing for extensibility, and continuing to empower our growing community of users and developers. In sum, this is how the Cytoscape Project is taking on the challenge of creating a network biology ecosystem for the 21st century.

## Data Availability

The original contributions presented in the study are included in the article/supplementary material, further inquiries can be directed to the corresponding author.
